# Sharing voxelwise neuroimaging results from rhesus monkeys and other species with Neurovault

**DOI:** 10.1016/j.neuroimage.2020.117518

**Published:** 2020-10-31

**Authors:** Andrew S. Fox, Daniel Holley, Peter Christiaan Klink, Spencer A. Arbuckle, Carol A. Barnes, Jörn Diedrichsen, Sze Chai Kwok, Colin Kyle, J. Andrew Pruszynski, Jakob Seidlitz, XuFeng Zhou, Russell A. Poldrack, Krzysztof J. Gorgolewski

**Affiliations:** a University of California, Davis and the California National Primate Research Center, Davis, CA 95616, USA; b Brain and Mind Institute, Western University, London, Ontario, Canada; c University of Arizona, Evelyn F. McKnight Brain Institute and Division of Neural Systems, Memory and Aging, Tucson, AZ, USA; d Netherlands Institute for Neuroscience, Royal Netherlands Academy of Arts and Sciences, 1105 BA Amsterdam, the Netherlands; e Shanghai Key Laboratory of Brain Functional Genomics, Key Laboratory of Brain Functional Genomics Ministry of Education, Shanghai Key Laboratory of Magnetic Resonance, Affiliated Mental Health Center (ECNU), School of Psychology and Cognitive Science, East China Normal University, Shanghai, China; f Division of Natural and Applied Sciences, Duke Kunshan University, Duke Institute for Brain Sciences, Kunshan, Jiangsu, China; g NYU-ECNU Institute of Brain and Cognitive Science at NYU Shanghai, Shanghai, China; h Shanghai Changning Mental Health Center, China; i Lifespan Brain Institute, Children’s Hospital of Philadelphia and University of Pennsylvania, Philadelphia, PA, USA; j Department of Psychology, Stanford University, Stanford, CA, USA

**Keywords:** Nonhuman primate, Neuroimaging, Data sharing, PET, fMRI, Animal

## Abstract

Animal neuroimaging studies can provide unique insights into brain structure and function, and can be leveraged to bridge the gap between animal and human neuroscience. In part, this power comes from the ability to com bine mechanistic interventions with brain-wide neuroimaging. Due to their phylogenetic proximity to humans, nonhuman primate neuroimaging holds particular promise. Because nonhuman primate neuroimaging studies are often underpowered, there is a great need to share data amongst translational researchers. Data sharing efforts have been limited, however, by the lack of standardized tools and repositories through which nonhuman neuroimaging data can easily be archived and accessed. Here, we provide an extension of the Neurovault framework to enable sharing of statistical maps and related voxelwise neuroimaging data from other species and template-spaces. Neurovault, which was previously limited to human neuroimaging data, now allows researchers to easily upload and share nonhuman primate neuroimaging results. This promises to facilitate open, integrative cross-species science while affording researchers the increased statistical power provided by data aggregation. In addition, the Neurovault code-base now enables the addition of other species and template-spaces. Together, these advances promise to bring neuroimaging data sharing to research in other species, for supplemental data location-based atlases, and data that would otherwise be relegated to a “file-drawer”. As increasing numbers of researchers share their nonhuman neuroimaging data on Neurovault, this resource will enable novel, large-scale, cross-species comparisons that were previously impossible.

## 1. Introduction

Understanding how biological processes in the brain give rise to complex psychological functioning remains a major aim for neuroscientists. Addressing this lofty goal promises to provide basic insight into human nature and revolutionize the treatment of psychiatric and neurological illness. The neuroscientific ecosystem includes numerous species, ranging from varied invertebrates, to rodents, monkeys, and, ultimately, humans. Each of these species provides unique and complementary opportunities for advancing scientific understanding. Nonhuman primate models play an important and unique role in this ecosystem, because they can provide an evolutionary bridge between mechanistic studies in other nonhuman species and the distributed neural activations we can observe in humans (Amaral, 2002; Kalin and Shelton, 2003; Fox et al., 2015; Nakahara et al., 2002). The relatively recent evolutionary divergence between monkeys and humans accounts for these species’ shared brain structure and composition, which includes a highly elaborated prefrontal cortex (Öngür and Price, 2000; Barbas, 1995; Barbas and Pandya, 1989). In part because of their large, homologous brains, there is an increasingly large community studying monkeys with the same non-invasive neuroimaging techniques that are commonly used in humans. Neuroimaging studies in nonhuman primates provide an important resource for comparative studies, as well as for understanding how targeted brain manipulations interact with distributed brain networks (see Klink et al., *Advances in Brain Perturbation and Non-Human Primate Imaging*, in this issue).

As nonhuman primate neuroimaging comes of age (PRIMatE Data Exchange (PRIME-DE) Global Collaboration Workshop and Consortium, 2020), there is an increased need to share findings. Unfortunately, nonhuman animal neuroimaging studies can have limited generalizability due to small sample sizes, and this problem is exacerbated by the limited availability of nonhuman primates and the cost of neuroimaging. Even in the best of cases, brain-wide neuroimaging studies are often underpowered to detect modest relationships (Cremers et al., 2017; Geuter et al., 2018; Button et al., 2013). For example, Yarkoni (2009) outlines a thought experiment, in which a study uses 20 subjects to study 10 regions in a distributed network, where each region is modestly correlated with the phenotype of interest, at *r* = 0.4. Correcting for multiple comparisons results in a threshold of *p* < .005 (*p* < .05, alpha-corrected for 10 comparisons). Power calculations reveal that the probability of detecting an effect in each ROI is only 13%. Thus, only approximately one ROI will reach significance, making the effects appear more localized than they actually are. Importantly, Yarkoni goes on to note that the effect size in significant ROIs is necessarily inflated, as the critical value for *p* < .005 ROI is *r* = 0.6. Therefore, localized effects identified through mass univariate statistical testing are guaranteed to be inflated relative to their actual size. This logic applies to voxelwise neuroimaging studies in which significant voxels are those where *signal* + *noise* > *threshold*. In underpowered studies, the threshold is greater than the true signal, and significant voxels represent regions where both signal and random noise are consistent with the hypothesis being tested. Together, these effects can lead researchers to believe they have identified strong, localized effects, even when the true signal is widely distributed.

Primate neuroimaging is no exception. Studies in humans suggest that the neural substrates of complex phenomena are more likely to be weak and distributed, as in the example above, than they are to be strong and localized (Cremers et al., 2017). This is likely to be the case in primates as well. In theory, researchers should all perform well-powered studies with hundreds of subjects. However, in light of the practical and ethical considerations inherent to nonhuman primate research, such studies are uncommon. Because of this, smaller studies remain important. Each study provides useful information for identifying subsets of a distributed network, and cannot be easily differentiated from studies that reveal a few regions with large effect sizes without replication. Therefore, aggregating across similar datasets is critical. This challenge is not necessarily easily addressed, given resource constraints, but in some cases, it can be circumvented by team science approaches, consortia, and other kinds of data sharing (Thompson et al., 2014; Mennes et al., 2013; PRIMatE Data Exchange (PRIME-DE) Global Collaboration Workshop and Consortium, 2020; Rosenberg et al., 2018). Here, we provide a complementary platform for researchers to share their voxelwise results, so that inferences can be made across studies.

Data sharing has important benefits for many scientific stakeholders, including scientists and funding agencies. Although it would be ideal to systematically reproduce all individual studies, there are many practical barriers that make direct replication difficult at-scale. The feasibility and cost of large-scale nonhuman primate work, for example, make meta-analysis even more important. Data sharing can encourage collaboration and connectedness between researchers with similar findings, and can enable research that isn’t feasible in a single laboratory. For example, although numerous studies have investigated the effects of maternal deprivation on the brains of young rhesus monkeys, researchers are unlikely to reach scientific consensus until they can inspect data in aggregate. Fortunately, meta-analytic techniques are particularly well-suited to areas where the neuroimaging measures can be readily collected and quantified using standardized techniques (e.g., resting and anatomical MRI data). To be unbiased, these meta-analytic techniques require more than the peak-coordinates and cluster-sizes that are typically reported, as every non-reported statistic is akin to an unpublished study in a standard meta-analysis (Lohmann et al., 2017). As such, it is critical to share results from nonhuman animal studies as completely, openly, and efficiently as possible.

Historically, even when nonhuman primate neuroimaging research is openly shared, it is often relegated to individual datasets shared through one-off websites and tightly-guarded file drawers. This limited and uncoordinated data sharing is suboptimal, leaving scientists to scour for available datasets. The field of human neuroimaging has begun to address this problem with online repositories that serve as centralized storage for various forms of neuroimaging data, e.g. NITRC (https://www.nitrc.org/), NIMH Data Archive (https://nda.nih.gov/), INDI (http://fcon_1000.projects.nitrc.org/), Neurovault (https://neurovault.org/), and many others. Each of these repositories provide structured frameworks for sharing neuroimaging data and overcoming these barriers to open science. Recently, researchers have taken aim at the barriers to fully open and reproducible nonhuman neuroimaging by arguing for homogenized data collection (Poirier et al., this issue; Baso and Schroeder, this issue) and by providing open resources for sharing analysis tools (Messinger et al., this issue) and raw data (Milham et al., 2018). Here, we provide a complementary tool that can be used to share unthresholded statistical maps and other voxelwise neuroimaging data, such as ROIs, atlases, and relevant quality-control images. More specifically, we describe an extension to Neurovault (Gorgolewski et al., 2015), a repository currently being used to share human neuroimaging results. Neurovault complements other repositories that are focused on sharing analysis tools (e.g., PRIME-RE, 2020 and the Neuroimaging Tools & Resources Collaboratory), and raw neuroimaging data (PRIME-DE).

Neurovault.org is a resource that allows researchers to upload and share their neuroimaging results, using a modern web-interface that allows easy uploading and efficient data-description. Neurovault.org, which is already available for human neuroimaging data, is intended to supplement the sharing of neuroimaging results by allowing researchers to share their regions of interest and unthresholded statistical maps, as well as relevant atlases and voxelwise quality-control estimates. It provides an excellent complement to other data-sharing resources. The human Neurovault resource has over 300,000 images across 4,500 different collections, demonstrating its popularity for sharing neuroimaging data. The existing interface allows users to interact with their data online and search for related datasets, and provides numerous meta-analytic tools that promise to aid in discovery. Until recently, Neurovault.org was limited because it was only able to accept human neuroimaging data that had been aligned to the MNI152 template-space. Here, we present an extension of Neurovault that allows sharing of multi-species neuroimaging data, including nonhuman primates.

## 2. Methods

**Fig. 1.**
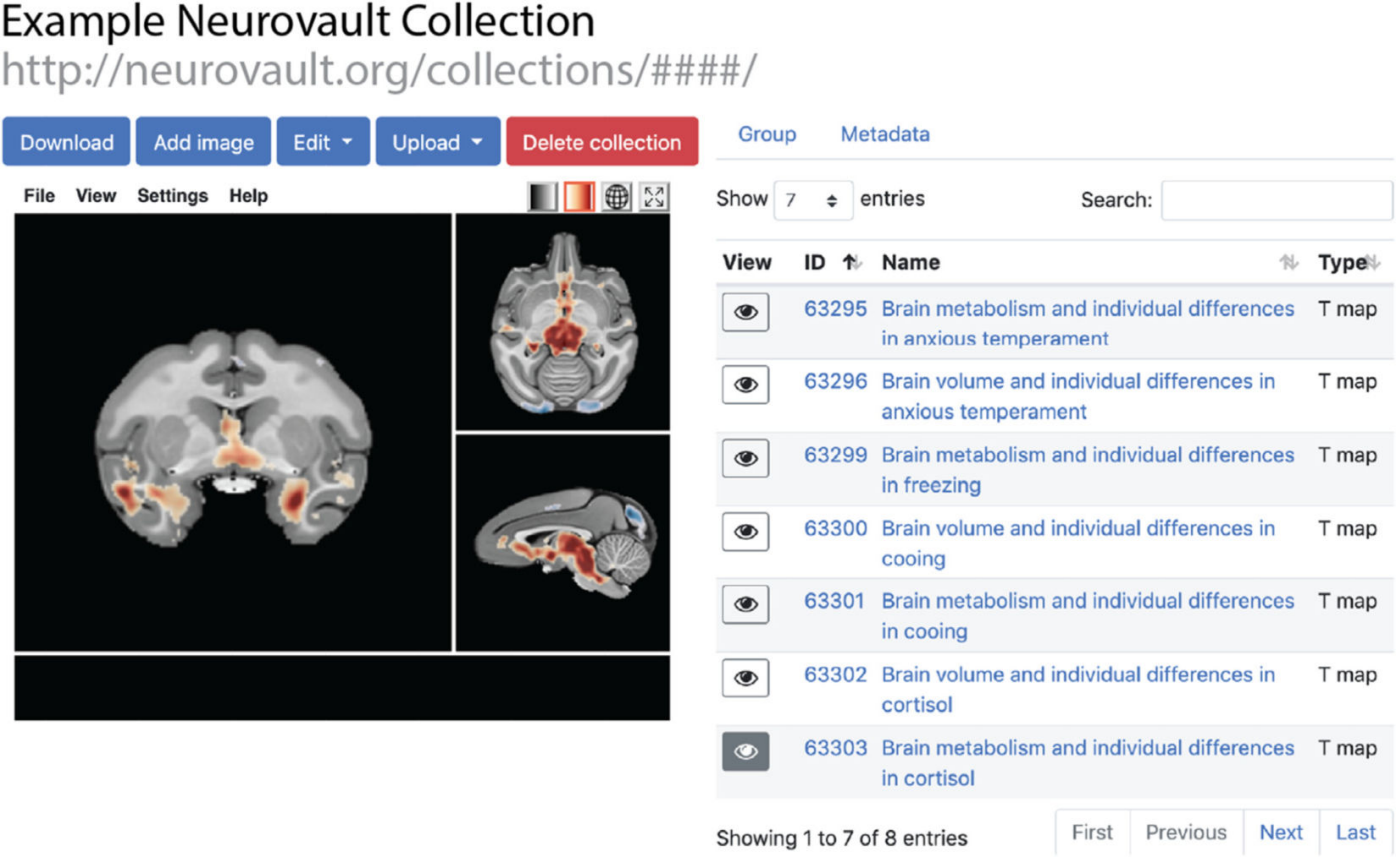
An example neurovault collection containing rhesus monkey data aligned to the NMT template (https://neurovault.org/collections/3739/). These data were reported, but not fully described in Fox et al. (2015). The selected image is the correlation between cortisol levels and regional brain metabolism measured with FDG in 592 young rhesus monkeys. The Neurovault interface allows interested researchers to further examine, threshold, and download these Supplementary Analyses.

Here, we present an updated version of Neurovault that supports other species and templates, including rhesus monkeys. With this new addition, Neurovault now allows researchers to add nonhuman primate statistical maps in NMT format (Fig. 1; Seidlitz et al., 2018).

Neurovault is already a leading repository for the storage of human neuroimaging data. It was designed to provide a user-friendly, interactive, browser-based visualization experience. Images can be easily and quickly uploaded to Neurovault, where they will be assigned a unique URL with an embedded viewer. Data uploaded to Neurovault may be published or unpublished. Published data can be linked to the DOI of the corresponding article, and a link to the Neurovault *Collection* can be included in the published article to provide interested readers with access to more information about significantly (and non-significantly) activated regions. Unpublished data can be shared publicly, or privately, in which case they remain inaccessible to anyone who does not have the unique URL.

Neurovault provides a user-friendly, well-documented method for uploading unthresholded statistical maps, mask files, parcellation maps, and any other voxelwise data aligned to the specified templates. Users can create an account and upload a *Collection* (generally corresponding to a study) with *Images* (generally corresponding to a voxelwise map). All uploads are supplemented by additional information inputted by the researcher with relevant study details, including data collection methodology (e.g., MRI acquisition details), data processing methodology (e.g. smoothing kernel), and subject information (e.g. number of subjects). These procedures have been used for human neuroimaging data sharing for over 5 years, and they are now available to researchers working with data from nonhuman animals. Data upload can be performed in less than 15 min. By adapting this resource, nonhuman animal researchers can benefit from years of experience and optimization of the Neurovault resource, as well as existing descriptions and documentation on Neurovault.org and from 3rd parties.

Neurovault hosts neuroimaging “ *Collections* “ that contain “ *Images* “. There is an opportunity to include meta-data for both *Collections* and *Images*. Species is implemented as the “ *Template* “ where the *Images* are aligned. Because the template is defined as *Image*-specific, researchers can upload *Collections* that include neuroimaging data from multiple species. This also provides a future-sensitive framework for cross-species and cross-template alignment. In addition to sharing neuroimaging results, we encourage authors to share voxelwise quality-control images. These images will allow interested parties to ensure their interpretations about unpredicted findings are reliable in the context of data acquisition.

**Fig. 2.**
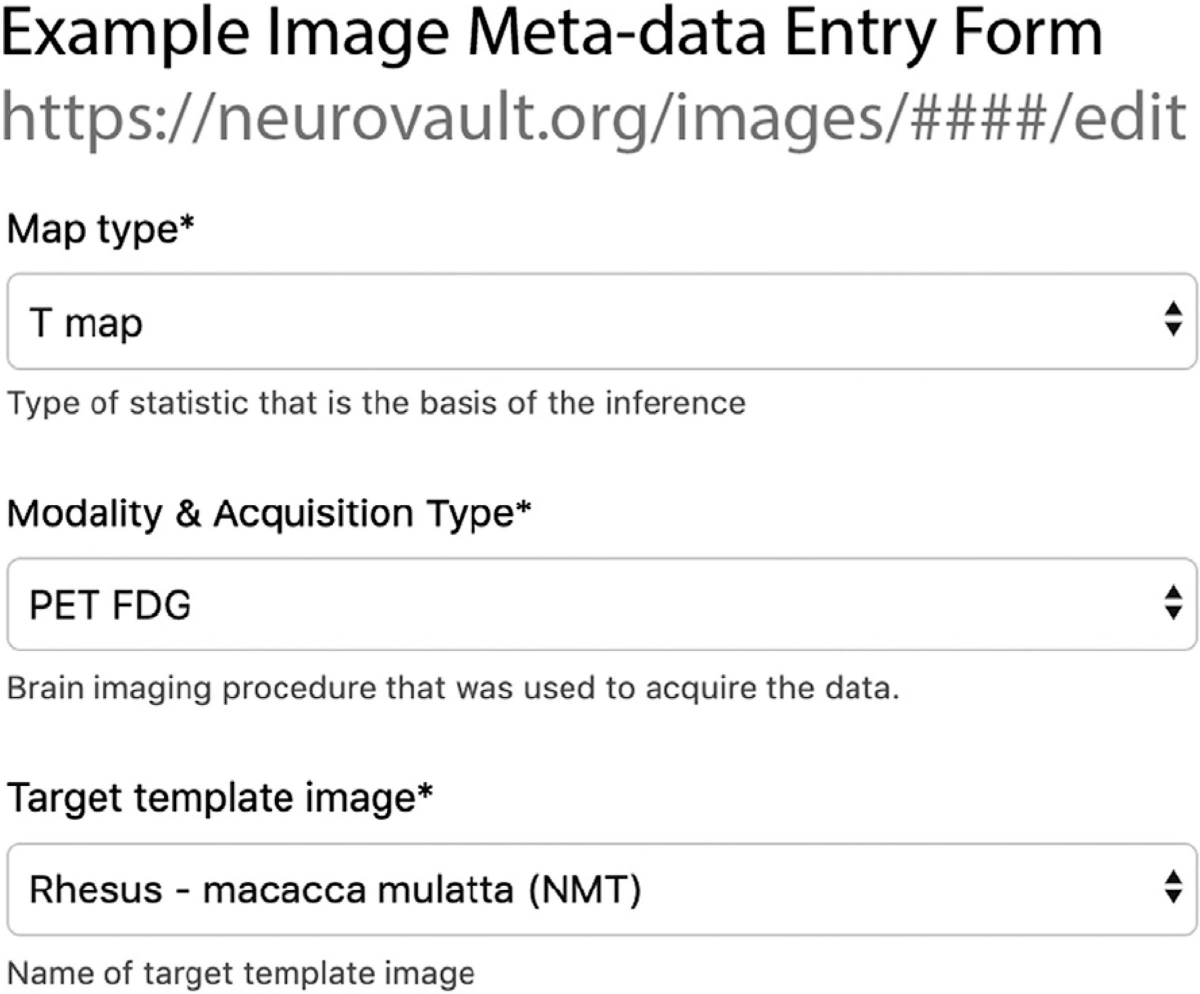
The image meta-data entry form that was used to specify the Target image template to which data were aligned. In this case, it was Rhesus – macacca mulatta (NMT). This is the only difference seen by the user when sharing nonhuman primate data, which allows the animal neuroimaging community to benefit from the work done to optimize Neurovault for human neuroimaging.

All user-input for nonhuman animal imaging studies is identical to the inputs that have been optimized for human researchers, except for a drop-down menu for each *Image* the user uploads, where they must specify the template-space/species to which their data are aligned (Fig. 2). For nonhuman primate neuroimaging, we chose NMT stereotaxic space because it provides a population based template across ages and sex, scripts for aligning data, and high-quality surface-maps (Seidlitz et al., 2018). Studies that have been aligned to other templates, such as the Yerkes19 (Donahue et al., 2016) and the INIA19 (Rohlfing et al., 2012), can be easily transformed to NMT space for upload to Neurovault. To facilitate transformation to NMT space from other standard spaces, Klink and colleagues developed the RheMAP package (Sirmpilatze and Klink, 2020; https://github.com/PRIME-RE/RheMAP). It provides pre-calculated transformations between NMT and other commonly used templates and code for the registration of individual anatomical images to numerous standard monkey template-spaces. The package is listed on the Primate Research Exchange (PRIME-RE, https://primere.github.io/) where researchers can also find links to template spaces and data-sharing solutions like Neurovault (see Messinger et al., *A Collaborative Resource Platform for Non-Human Primate Neuroimaging*, in this issue). Importantly, fields for metadata that the experimenter does not have can be left blank; the required fields are *Collection Name* and *Description, Image Name, Type, Modality*, and *Target Template*. There is the opportunity to include other information, including *N, task*, analysis software, and *data collection parameters.* This information is recommended, but not required.

Although Neurovault is designed for public data sharing, researchers can also share private *Collections*, which can only be seen by researchers with the direct link to the data. This provides an excellent method for data sharing for ongoing studies across sites and an easy way to convert a private data-set to a publically shared one when a study is finished or the results are published. All public data uploaded to Neurovault.org receive unique Compact Identifiers (Wimalaratne et al., 2018). A tutorial blog, *Getting Started with Neurovault*—which walks users through the process of uploading, interacting with, and searching for images—is available here: https://github.com/DanHolley/neurovault_blog/blob/master/README.md.

## 3. Results

We have already begun to upload and share data from rhesus monkeys neuroimaging experiments. Many of these images come from previously published manuscripts e.g. (Fox et al., 2015, 2018). To showcase the kinds of data that can be shared on Neurovault.org, we now highlight a few example datasets that demonstrate how we can use this resource to supplement published papers; empty our “file drawer”; and share other voxelwise resources, such as anatomical maps.

### Supplementing published papers:

For many neuroimaging papers, there are multiple ways to look at the data, and many ancillary analyses that could be run. Such analyses are often only briefly mentioned and relegated to a line or two in the published article. Although these data may be of interest to other researchers, the publication format prevented their complete data sharing. For example, a focus of Fox et al. (2015) was a correlation between heritable brain metabolism and variation in a composite measure of anxious temperament. The manuscript did not focus on correlations with individual components of anxious temperament that made-up the composite (freezing, decreased vocalizations, and cortisol), nor did it fully describe the correlations between anxious temperament and regional brain volume. Although the major points of these analyses were reported, they were relegated to the supplemental material and detailed descriptions of the findings were not reported. Now that these voxelwise associations are shared on Neurovault (https://neurovault.org/collections/3739/), this information is available to the public (e.g., cortisol and brain metabolism shown in Fig. 1). Importantly, this approach can be used to share other supplemental data that can help inform the main manuscript, including relevant quality-control data (e.g. voxelwise signal-to-noise ratio estimates).

### Unpublished data:

**Fig. 3.**
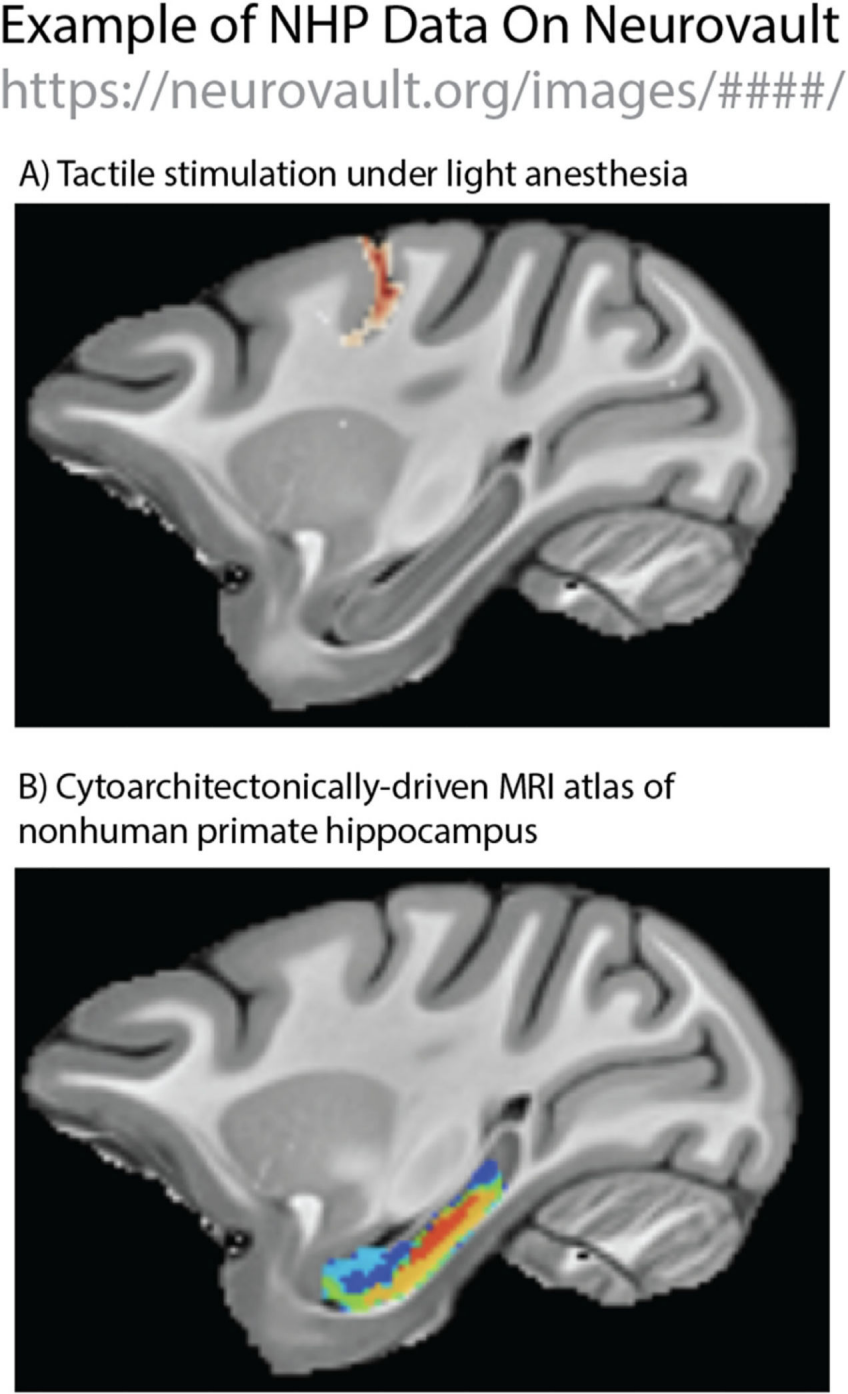
Example rhesus monkey neuroimaging data from Neurovault.org. A) *Unpublished Data:* Tactile stimulation of individual fingers vs. rest during fMRI of a lightly anesthetized animal (isoflurane ~0.75%; https://neurovault.org/collections/3864/). These data may be useful to other researchers interested in developing test procedures in lightly anesthetized animals. B) Location-based Data: A cytoarchitectonically-driven MRI atlas of rhesus monkey hippocampus (https://neurovault.org/collections/4083/). Like all Neurovault data, these can be downloaded and integrated into ongoing analyses.

Neurovault is not limited to data that accompany published manuscripts. Many labs have data that were difficult to interpret, were not further investigated after initial pilots, or did not reach the threshold for publication for some other reason. In many cases, these data may still be of interest to the neuroimaging community. For example, Arbuckle, Diedrichsen, and Pruszynski have shared functional scans from a male rhesus macaque that received passive stimulation of individual fingers under low (~0.75%) isoflurane anesthesia (Fig. 3 A; https://neurovault.org/collections/3864/). These data may provide important preliminary information that can guide other researchers, which would otherwise have been lost in the “file-drawer”. Importantly, sharing data on Neurovault does not prevent the researchers from publishing their data at a later date. It simply speeds up the timeline with which these data can be used by other scientists to forward the field.

### Location-based resources:

Sharing resources voxelwise resources like atlases and maps, can be onerous and difficult. Even once a researcher has painstakingly created an atlas of their target brain region and defended it in publication, it is not always clear how to best share this resource with the community. This can become a particularly thorny problem as lab personnel change, and file-servers are replaced (though, see: NITRC.org). Neurovault.org provides a unified place for sharing and visualizing location-based resources, including masks and regional atlases. For example, Kyle et al. (2019), provided a cytoarchitectonically defined atlas of nonhuman primate hippocampus (Fig. 3 B). This atlas is now shared and stored on Neurovault (https://neurovault.org/collections/4083/), ready to be downloaded by interested researchers.

### Uniquely nonhuman animal datasets:

Nonhuman animals can enable invasive studies that are not possible in humans. Neuroimaging can provide a critical link between localized mechanistic studies, and the distributed patterns of brain activation commonly observed in human neuroimaging studies (see *this issue*). Neurovault provides a unique opportunity to share distributed neuroimaging results after mechanistic manipulation, such as lesions, optogenetics, DREADDs, and alterations of gene expression (https://neurovault.org/collections/4162/). Fox et al. (2019), for example, identified changes in a distributed brain network after regional gene manipulation. These kinds of data provide critical information about the potential source of the distributed brain alterations observed in human studies.

## 4. Discussion

Nonhuman primate neuroimaging studies frequently struggle to reach the number of animals required to demonstrate reproducible results (though see: (PRIMatE Data Exchange (PRIME-DE) Global Collaboration Workshop and Consortium, 2020; Oler et al., 2010; Fox et al., 2015, 2018). Although NHP neuroimaging studies are typically performed with small samples, meta-analytic techniques can be used to gain additional insights. Combining unthresholded voxelwise statistical maps across studies might provide the necessary power for new discoveries that would not have been possible in any single sample. The Neurovault framework allows researchers to upload their neuroimaging results, thus enabling cross-site, multi-study, voxelwise meta-analyses that are likely to produce estimates of effect-size that are more reliable and realistic than any one study alone.

Importantly, Neurovault is designed to share neuroimaging results, not raw, unprocessed, neuroimaging data. In this way, Neurovault is intended to complement existing frameworks for sharing raw data, such as PRIME-DE (PRIMatE Data Exchange (PRIME-DE) Global Collaboration Workshop and Consortium, 2020), NeuroImaging Tools & Resources Collaboratory (NITRC), and the National Institute of Health’s Data Archive (nda.nih.gov). Accumulating data across imaging sites will require stimuli, acquisition, and analysis standardization that can only be achieved through thoughtful collaboration and the sharing of raw data. For example, when the large retinotopy dataset of the human connectome project (Benson et al., 2018) was shared, re-analysis revealed retinotopic information in the cerebellum (Van Es et al., 2019) and default mode network (Szinte and Knapen, 2020). Importantly, the statistical maps resulting from these re-analyses can, and should, be shared on Neurovault so that these results can be more thoroughly studied.

Data sharing resources can enable increasingly integrative data analytic techniques and provide a mechanism for sharing additional analyses. In humans, Neurovault has already enabled integration between neuroimaging data and gene expression data, allowing users to identify genes that are most expressed in the brain regions associated with their neuroimaging data (Gorgolewski et al., 2014; Fox et al., 2014). The non-human primate neuroimaging community is well-poised to benefit from these kinds of resources, which are facilitated by the Neurovault framework. For example, as researchers work toward cross-species spatial co-registration (e.g., Eichert et al., 2020; Xu et al., 2019), these benefits can be immediately applied to the Neurovault database so that functional maps can be compared across species.

It must also be noted that combining datasets poses challenges that can lead to unintentional misuse. For that reason, Neurovault users should be aware of certain considerations. For example, similar studies often have different acquisition parameters, or feature nuanced differences in design or setting that influence their results. Decisions made at each step of the fMRI pipeline can change the interpretation of the data (Botvinik-Nezer et al., 2020; Carp, 2013; Soares et al., 2016). While this is true in standalone studies, the potential for error is compounded when combining datasets, in part because the researchers who do so are inherently less familiar with aspects of acquisition, study design, processing, etc., than the originators of the data. Nevertheless, there remains a substantial benefit to accumulating data from multiple studies in an attempt to identify reliable neuroimaging results. To address this, Neurovault encourages researchers to share details of their acquisition protocols and processing pipeline by providing standardized opportunities to document these data. We highly encourage Neurovault users to be as descriptive and complete as possible when submitting their images, and to keep these challenges in mind when combining Neurovault datasets into their own analyses.

In addition to our focus on primate neuroimaging, we have extended the Neurovault framework to provide flexibility for researchers in other species. We have already extended Neurovault to host data from studies in C57Bl/6 J mice (Dorr et al., 2008). By submitting their template and changing a few lines of code, Neurovault is ready to expand, and provides an opportunity for researchers working with other species; for example rats, rabbits, dolphins, etc. Increasing the phylogenetic diversity of data shared on Neurovault will increase the opportunities for cross-species and evolutionary comparisons. It is precisely the functional and structural neuroimaging maps that can be shared on Neurovault that can facilitate multi-modal registration techniques. As such, the open sharing of these data can be of use outside of one’s own lab and help guide our understanding of brain organization across species.

Ultimately a great challenge that will prevent society from reaping the benefits of animal models will be our limited ability to translate knowledge to humans. Studies in nonhuman animals are critical for understanding how mechanistic molecular and cellular insights gleaned from rodents alter the distributed neural systems known to underlie complex function in humans. Our rhesus monkey Neurovault resource can facilitate cross-species translation by providing a common place to identify cross-species neuroimaging studies addressing conceptually similar topics, enabling integrated cross-species alignment tools as they become available, and an easy-to-use, public-facing site that allows researchers to explore neuroimaging data from multiple species with minimal barriers to entry. The future of nonhuman neuroimaging will require reliable data sharing and use across research labs, as enabled by Neurovault. We are pleased to offer this tool to the community, and look forward to the innovation its openly-shared data promise to enable.

## Data Availability

All data and code is freely available online. Data is available on Neurovault.org, through the links provided in the manuscript. Neurovault is an open-source project that is available on github (https://github.com/NeuroVault/NeuroVault).

## BOX 1. Neurovault for other species and/or other template spaces

Though our focus was on creating a mechanism for sharing rhesus monkey neuroimaging data, the needs and reasons for extending Neurovault certainly extend to other species. To meet this need, we created an extensible Neurovault framework for incorporating other species or template-spaces. To allow Neurovault to accept data that is aligned to a new template, including those from other species, researchers can initiate a pull-request to the Neurovault github repository by adding an entry to the POSSIBLE_TEMMPLATES dictionary (Fig. B1). To demonstrate the power of this approach, we have already included a template for adult C57Bl/6 J mice from Dorr et al. (2008).
